# Involvement of high school teachers in Health Promoting School program in selected township, Yangon Region, Myanmar: A cross-sectional mixed methods study

**DOI:** 10.1371/journal.pone.0270125

**Published:** 2022-06-16

**Authors:** Kaung Myat Naing, Ye Minn Htun, Kyaw Myo Tun, Tun Tun Win, Htein Lin, Than Tun Sein

**Affiliations:** 1 Outpatient Department, Defence Services Liver Hospital, Yangon, Myanmar; 2 Department of Prevention and Research Development of Hepatitis, AIDS and Other Viral Diseases, Health and Disease Control Unit, Nay Pyi Taw, Myanmar; 3 Department of Preventive and Social Medicine, Defence Services Medical Academy, Yangon, Myanmar; 4 Department of Anthropology, University of Yangon, Yangon, Myanmar; GITAM College of Pharmacy: GITAM Institute of Pharmacy, INDIA

## Abstract

**Background:**

Schools provide a big opportunity for promoting the student’s health, life skill, and behavior. Teachers play a fundamental role in the promotion and successful implementation of school health services. This study aimed to assess the level of involvement in the Health Promoting School program and its associated factors and to explore the benefits and barriers to involvement among high school teachers in Myanmar.

**Methods:**

A mixed methods explanatory sequential study was conducted among 194 high school teachers in Thanlyin Township, Yangon Region, Myanmar, from June to August 2020. Quantitative data were collected with the pretested structural questionnaire and analyzed by Chi-square tests and Fisher’s exact tests. A qualitative strand was added by conducting in-depth interviews (n = 15, five teachers from each level of involvement: poor, medium, and good), analyzed by thematic content analysis.

**Results:**

Of the 194 teachers, 23.7% had a good level of involvement in the Health Promoting School program. The factor associated with involvement in Health Promoting School program were age (p value < 0.001), duration of services (p value = 0.001), and a number of accomplished training-related school health (p value = 0.008). Qualitative data revealed that improvement of the health knowledge and awareness on health problems, the progress of healthy behaviors, development of physical and mental health, prevention of the disease spread, achievement of healthy and productive learning environment, and development of academic achievement were major benefits of teachers’ involvement. Moreover, the main barriers to involvement were insufficient materials and human resources, time constraints, incompetence of the teachers, poor cooperation of school health partnerships, and insufficient awareness of parents.

**Conclusions:**

The proportion of good involvement in the Health Promoting School program among high school teachers was low in this study area. Providing sufficient human resources and material, conducting the on-the-job and refresher training, enhancing parent-teacher cooperation, and strengthening the community partnerships were crucial to improve the level of involvement and reduced the barriers for the achievement of the Health Promoting School program.

## Introduction

Education, a primary driver of sustainable development, improves the skills, values, and attitudes that enable people to achieve healthy and fulfilled lives [1]. Health is fundamental to education and so, poor health can give a detrimental effect on school attendance and academic performance [2]. Schools provide a chance for the students to acquire knowledge in various fields of education and healthy living throughout their entire lives. Schools are also increasingly seen as an important setting not only for promoting long-term educational attainment but also for supporting the health and well-being of students, their parents or caregivers, and the local community [3–5].

The 1.2 billion adolescents in the world today represent more than one-sixth (18%) of the global population. Globally, over 80% of children in the lower secondary school age are enrolled in school where they spend one-third of their time. It makes the school a unique setting for preventive interventions, and school years as an important period to establish healthy behaviors that will contribute to a lifetime of health promotion [6]. Infectious diseases, accidents and injuries, nutritional disorders, substance abuse, and emotional disturbances were the common health problems encountered among school children [7]. As the challenges, global mortality and morbidity estimates in children and adolescents suggest that school-age children have significant needs for health promotion, prevention, and health care services [6, 8, 9].

As the school is a place where education and health programs can have their greatest impact on the students at influential stages in their lives, it provides a great opportunity for promoting the student’s health [7, 10]. School health services are part of the whole school approach that is promoted by the World Health Organization (WHO) through the Global School Health Initiative launched in 1995 [11]. It aims to promote and maintain the health of school children so as to give them a good start in life [12]. The initiative supports the countries to implement the four pillars for Health Promoting School (HPS) such as HPS policies, safe and healthy learning environment, skill-based health education, and school-based health and nutrition services [13].

A HPS is a school that is constantly strengthening its capacity as a healthy setting for living, learning, and working [4, 14]. By creating HPS, a healthy school movement would seek to improve school achievement; promote positive development and healthy behaviors; create school environments that promote lifelong individual, family, and community health; and focus on prevention of chronic illness, violence, and problems of sexual and mental health [3, 6]. The health of the students contributes critically to education performance and school enrolment. The dual benefits regarding the implementation of HPS are directing to achieve “health for all” and “education for all” [15]. Therefore, the HPS acknowledges the close connection between health and education [16].

School teachers have the capability of influencing the future, knowledge, attitude, and behavior of school children. They are considered to be an important target group for various health educational activities with the underlying objective of inculcating healthy lifestyle practices into children for a lifetime [10]. In addition, school teachers are the key to establishing a welcoming climate of open communication and partnership between parents and students [17–19]. The involvement of school teachers in the HPS activities can contribute to an enhancement of action competencies on healthy living with professional development [20].

In Myanmar, since 1920, the Ministry of Health and Sports (MOHS) in collaboration with the Ministry of Education (MOE) has implemented school health services. The MOHS and the MOE of Myanmar were jointly reforming the school health program with a greater emphasis on comprehensive health services and health literacy, environmental health and sanitation, school-based disease control and mental health as well as injury and violence prevention. In 1996, Myanmar has adopted the HPS program with the concept of WHO School Health Initiatives. As stated by the report of Myanmar Health Management Information System, in 2015, only 38.8% of schools covered the HPS activities [15]. In 2016, Myanmar Global School-based Student Health Survey validated that the prevalence of unhealthy behaviors such as unhealthy dietary habits including eating junk foods (46%) and drinking carbonated soft drinks (45%), physical inactivity (30.2%), alcohol drinking (4.3%), and using tobacco (6.6%) increased among 13–17 year students [21].

This survey also revealed that poor nutritional status, poor dietary habits and low levels of physical activity, high levels of tobacco use, relatively high incidence of injuries, physical violence and bullying, and poor mental health indicators were the worrisome findings among schoolgoing adolescents in Myanmar [21]. These findings pointed out the need for comprehensive school health activities by related ministries and all stakeholders. Therefore, adolescent students in Myanmar were in increased need of focused interventions to reduce exposure to risk factors of non-communicable diseases, nutritional problems, and social and mental health issues through multisectoral approaches.

Enhancing personal knowledge and involvement of the school teachers in school health activities are essential in ensuring the effectiveness and overall success of the HPS program. Although school teachers have the full authority for the promotion and successful implementation of the school health services, challenges remain in the involvement of school teachers in the HPS program with the contributing factors of inadequate resources for school health services, insufficient facilities in schools, low accessibility of training related school health services, low knowledge, and poor awareness and involvement on school health services [12, 22–24]. Additionally, teachers face a variety of challenges in their social interactions with students, parents, and health professionals and the differences in culture, personal characteristics, and school-related issues [25].

For the involvement of school teachers in the HPS program to be satisfactorily improved in Myanmar, it is necessary to assess the level of involvement in the HPS program and its related factors among high school teachers. However, there is limited information regarding the involvement of school teachers in the HPS activities in Myanmar. One cross-sectional study investigating the reported practices on specified school health activities was conducted in primary school teachers [26]. This study, therefore, aimed to assess the level of involvement and to explore the benefits and barriers to involvement in the HPS activities among high school teachers in Myanmar.

## Methods

### Study design

The study was adopted as a mixed methods explanatory sequential design incorporating a cross-sectional survey of high school teachers and in-depth interviews of selected participants [27]. Mixed methods research is useful in understanding contradictions between quantitative results and qualitative findings [28] and typically capitalize on data reflecting individual lived experiences [29].

### Study area, period, and population

In Myanmar, there were 38.7% of schools with HPS activities in 2016. More discrepancy among states and regions was occurred in HPS activities and it was 78.8% in Yangon Region [30]. This study was conducted in all high schools of selected township, Thanlyin, Yangon Region, Myanmar, from June to August 2020. Thanlyin Township (16° 45’ 57.06’’ N, 96° 15’ 5.38’’ E) is located 10.42 kilometers far from Yangon City, where is an industrial and commercial center of Myanmar. There were 194 teachers in a total of 14 high schools in Thanlyin Township. The target population of the study was the high school teachers who were assigned as government staff under the MOE.

### Sample size determination and sampling procedures

For the quantitative strand, the sample size was determined by using the single population proportion formula [31], with an assumption of 8% margin of error, 95% confidence interval, and 52.6% of teachers with good practice scores in school health activities [26]. The minimum sample size including a 10% non-response rate was 165 high school teachers. All high school teachers of Grade 10 and 11 in 14 high schools were included in this study and therefore, the final sample size was 194. The teachers who were in training or on leave at the time of data collection, and those who were in private schools, and those who were not willing to give informed consent were excluded.

For the qualitative strand, In-depth Interviews (IDIs) were conducted in the Burmese language to underscore and contrast opinions, benefits and barriers on the HPS program. Based on the quantitative data analysis, a total of fifteen teachers (five teachers from each level of involvement: poor, medium, and good) were selected by simple random sampling.

### Data collection tool and procedures

#### Face-to-face interviews

The quantitative data were collected by face-to-face interview method using the structured questionnaire. The questionnaire was constructed to assess the level of knowledge, attitude, and involvement on the HPS program. It was developed based on an extensive literature review [26] and also adopted from the main issues of school health by MOHS, Manual for School Health, 2019 [32], and Myanmar National Comprehensive School Health Strategic Plan (2017–2022) [15]. The questionnaires were comprised of four parts (**S1 File**): personal characteristics, knowledge, attitudes, and involvement on the HPS program. The first part was the personal characteristics (i.e., sex, age, services, education, training, and school location for school health). The second part consisted of 20 knowledge questions towards activities of the HPS with the option of “yes”, “no”, and “don’t know”. The scoring for the knowledge questions was one points for correct response and zero point for incorrect and “don’t know” responses.

As the third part, attitudes of the high school teachers towards HPS program was assessed with 27 statements including negative and positive aspects. A five-point Likert’s scale was used for the scoring of the attitude statements: strongly agree (five points), agree (four points), uncertain (three points), disagree (two points), strongly disagree (one point) for positive statements, and reverse scoring for the negative statements. The fourth part comprised 43 questions to assess the involvement of high school teachers on the HPS program. The scoring was one point for each involvement in multiple responses and the more frequently involved teachers got more scores in a single response.

The English questionnaire was translated into the Burmese language, local language, and translated back to English. Before the data collection, content validation was performed based on expert review. A pretest was conducted among 15 high school teachers (5% of the sample size) in No.5, Basic Education High School, Mingaladon to assess the reliability. The pilot study data were not included in study results but served for adjusting minor modifications to the questions. The reliability was assessed by calculating the Cronbach’s α coefficients, which were found to be satisfactory for the second part (0.81 for knowledge), the third part (0.73 for attitudes), and the fourth part (0.85 for involvement) of the questionnaires. After refining according to pretest results, the questionnaires with the Burmese language were used for data collection. The two interviewers were trained for data collection, however, they were not informed about the correct answers in order to avoid interviewer bias during data collection. Before the data collection, the interviewers explained the purpose and procedure of the study to the participants. The face-to-face interviews were performed in the teachers’ restroom after receiving the written informed consent from all participants.

#### In-depth interviews

The IDIs aimed to explore the benefits and barriers to involvement in the HPS program. Following the explanatory sequential design, and reviewed by literature [26, 27], the nine domains of HPS activities in school were used as main themes of IDIs guide: 1) school-based health literacy promotion, 2) healthy environments, 3) prevention and control of both communicable and non-communicable diseases, 4) nutrition promotion and food safety, 5) school health services, 6) physical education, fitness, and sports, 7) counseling, and social support, 8) community outreach, and 9) training and research (S2 File) [15, 33]. The subthemes were developed and structured to explore opinions, benefits, and barriers of involvement in each HPS activity. The informed consent for the interview, permission to record with a voice recorder, and agreement to use the information recorded as direct quotes were requested from the teachers. After receiving informed consent from all participants, IDIs were conducted in a teachers’ restroom during class break times. The length of the IDIs was a range of 30 to 45 minutes, and all interviews were audio-recorded for transcription purposes.

### Operational definitions

School health training is the act of increasing the knowledge and skill of teachers for performing the school health services. Life skill education was a group of psychosocial competencies and interpersonal skills that help people make informed decisions, solve problems, think critically and creatively, communicate effectively, build healthy relationships, empathize with others, and cope with and manage their lives in a healthy and productive manner [34]. Knowledge was a consciousness or understanding of the activities related HPS program. The total scores of knowledge were categorized as low (< 25^th^ percentile), medium (25^th^ -75^th^ percentile), and high (> 75^th^ percentile). The attitudes were a way of feeling, beliefs, and behaviors towards a HPS program by the high school teachers, and the summarized attitudes scores were categorized as three: negative (< 25^th^ percentile), neutral (25^th^ -75^th^ percentile), and positive (> 75^th^ percentile). Involvement of the high school teachers was defined as practices or performance of activities in the HPS program. Based on the overall scores, and the level of involvement was considered as poor (< 25^th^ percentile), medium (25^th^ -75^th^ percentile), and good (> 75^th^ percentile).

### Data quality control

The two interviewers were provided the two-day training related to the clarity of the questionnaire, objectives of the study, confidentiality of information, and voluntary participation (informed consent) of the participants. The daily recorded data of IDIs were transferred to storage devices after data collection to ensure data security and back-up on a regular basis. Supervision was taken throughout the data collection period by the supervisor. The principal investigator closely monitored the interviewers for incompleteness, non-accuracy, and inconsistency of data.

### Statistical analysis

Data were entered into the Microsoft Excel sheet. After checking the errors, data were transferred into IBM SPSS Statistics for Windows, Version 25.0 (Armonk, NY: IBM Corp) for analysis. The normality of the continuous data was viewed by using the Kolmogorov-Smirnov test and the data distribution was non-normal. Categorical variables were presented as frequency (percentages) and continuous variables were described as median (interquartile range, IQR). Chi-square (χ^2^) test and Fisher’s exact test were used to assess the associations of personal characteristics, knowledge, and attitudes with outcome variable, level of involvement in the HPS program. All p values were considered as the level of statistical significance was at <0.05.

All IDIs were transcribed verbatim and analyzed thematically. Verbatim records were translated into English. The English transcripts were coded and performed the quality check on these transcripts to ensure consistency between audio recordings and transcripts. The deductive codes were comprised in the framework analysis process. After making the sense of the data, initial codes were drawn based on the research question and from the existing literature of HPS program which was hypothesized on subthemes of nine domains. The data were coded and then charting was done to arrange the data into appropriate subthemes of nine domains. The excel spreadsheet was used in the manual coding process.

### Ethical consideration

The study was approved by the Ethical Review Committee of the Post Graduate Board of study, Defence Services Medical Academy, and all methods were conducted with the provisions of the ethical approval. Before the data collection, the participants have explained the purpose, procedure, and effectiveness of the study. Then, the documentation involved the use of a written consent form containing all the information to be disclosed and signed by the participant. Both the face-to-face interviews and IDIs were conducted after receiving informed consent from all participants. The interviewing process was secured by choosing a quiet teachers’ restroom. All participants’ data was anonymized during the reporting of research findings.

## Results

The mixed methods typically capitalizes on data reflecting individual lived experiences and provides more policy-relevant insights [35]. Considering the results from the perspective of the participants, it can focus on the needs and priorities of the study population [29].

### Quantitative strand

#### Personal characteristics of high school teachers

A total of 194 high school teachers participated in this study and the personal characteristics of high school teachers were described in Table 1. Of all high school teachers, 184 (94.8%) were females and 10 (5.2%) were males. Teaching, one of the female-dominated professions in Myanmar might be due to that the women are more likely to have traits of caring for the children and they have sociological perspectives such as nurture and cultural surroundings. The median (IQR) age of high school teachers was 36 (18.25, 28.00–46.25) years with a minimum of 21 years and a maximum of 59 years. Most of the high school teachers, 159 (81.9%), were achieved B.Ed. degree. The median (IQR) duration of services was 13 (15.00, 5.00–20.00) years with a range of 1 to 40 years and 76 (39.2%) high school teachers had lower than 10 years of services duration. Among the school health services-related training, 184 (94.8%) high school teachers accomplished the teacher training course. Regarding the number of accomplished training for school health services, 77 (39.7%) high school teachers were accomplished one training and 64 (33.0%) accomplished three trainings. As a geographical background of schools, 138 (71.1%) high school teachers were from urban schools.

**Table 1:** Personal characteristics of high school teachers.

Variables		n (%)	Median (IQR)	Minimum, Maximum
**Sex**				
	Male	10 (5.2)		
	Female	184 (94.8)		
**Age (years)**			36.00 (18.25, 28.00–46.25)	21, 59
	< 40	115 (59.3)		
	40–49	44 (22.7)		
	≥ 50	35 (18.0)		
**Education**				
	B.Sc. or B.A.	10 (5.2)		
	B.Ed.	159 (81.9)		
	M.Sc. or M.Ed. and above	25 (12.9)		
**Duration of services (years)**			13.00 (15.00, 5.00–20.00)	1, 40
	< 10	76 (39.2)		
	10–19	66 (34.0)		
	≥ 20	52 (26.8)		
**Accomplished training for school health (multiple response)**				
	Teacher training course (TTC)	184 (94.8)		
	Refresher course	87 (44.8)		
	Life-skills education	104 (53.6)		
**Number of accomplished training for school health**				
	One training	77 (39.7)		
	Two trainings	53 (27.3)		
	Three trainings	64 (33.0)		
**School location**				
	Rural	56 (28.9)		
	Urban	138 (71.1)		

#### Level of knowledge, attitudes, and involvement in the Health Promoting School program among high school teachers

As shown in Table 2, the median (IQR) score of knowledge about HPS was 77.00 (6.25, 73.75–80.00) with a range of 43 to 87, and 38 (19.6%) high school teachers had a high level of knowledge. For the level of attitudes towards the HPS program, the median (IQR) score was 105.00 (11.25, 100.00–111.25) with 82 minimum scores and 129 maximum scores. Among the total high school teachers, 48 (24.7%) high school teachers had positive attitudes. The median (IQR) score of involvement in HPS activities was 95.00 (16.25, 85.75–102.00) with a range of 51–117 and 46 (23.7%) high school teachers had good involvement.

**Table 2:** Knowledge, attitudes and involvement of Health Promoting School program among high school teachers.

Variables		n (%)	Median (IQR)	Minimum, Maximum
**Level of knowledge**			77.00 (6.25, 73.75–80.00)	43, 87
	Low	48 (24.7)		
	Medium	108 (55.7)		
	High	38 (19.6)		
**Level of attitudes**			105.00 (11.25, 100.00–111.25)	82, 129
	Negative	42 (21.6)		
	Neutral	104 (53.6)		
	Positive	48 (24.7)		
**Level of involvement**			95.00 (16.25, 85.75–102.00)	51, 117
	Poor	48 (24.7)		
	Medium	100 (51.5)		
	Good	46 (23.7)		

#### Factors associated with the level of involvement in the Health Promoting School program among high school teachers

The associated factors of involvement in HPS program among high school teachers were described in Table 3. Aged 50 years and older high school teachers with good involvement were more than other aged categories and the association of age of high school teachers with the level of involvement was statistically significant (X^2^ = 20.639, p value < 0.001). The high school teachers with 20 years and more service duration were more likely to have good involvement than their counterparts. The association between duration of service and level of involvement in HPS program was statistically significant (X^2^ = 18.576, p value = 0.001). The high school teachers who accomplished three trainings regarding school health services were more likely to have a good level of involvement than those who accomplished one and two training. The association between the number of accomplished training and level of involvement was statistically significant (X^2^ = 13.796, p value = 0.008).

**Table 3:** Factors associated with level of involvement in Health Promoting School activities among high school teachers.

Variables		Total n (%)	Level of involvement			Chi-square (or) Fisher’s exact	p value
			Poor n (%)	Medium n (%)	Good n (%)		
**Sex**							
	Male	10	2 (20.0)	7 (70.0)	1 (10.0)	1.294	0.582*
	Female	184	46 (25.0)	93 (50.5)	45 (24.5)		
**Age (year)**							
	< 40	115	34 (29.6)	63 (54.8)	18 (15.7)	20.639	<0.001
	40–49	44	8 (18.2)	26 (59.1)	10 (22.7)		
	≥ 50	35	6 (17.1)	11 (31.4)	18 (51.4)		
**Education**							
	B.Sc./B.A.	10	1 (10.0)	8 (80.0)	1 (10.0)	8.487	0.062*
	B.Ed.	159	37 (23.3)	79 (49.7)	43 (27.0)		
	M.Sc./M.Ed. and above	25	10 (40.0)	13 (52.0)	2 (8.0)		
**Duration of service (year)**							
	< 10	76	24 (31.6)	46 (60.5)	6 (7.9)	18.576	0.001
	10–19	66	14 (21.2)	32 (48.5)	20 (30.3)		
	≥ 20	52	10 (19.2)	22 (42.3)	20 (38.5)		
**Number of accomplished training for school health**							
	One training	77	24 (31.2)	42 (54.5)	11 (14.3)	13.796	0.008
	Two trainings	53	12 (22.6)	31 (58.5)	10 (18.9)		
	Three trainings	64	12 (18.8)	27 (42.2)	25 (39.1)		
**School location**							
	Rural	56	16 (28.6)	30 (53.6)	10 (17.9)	1.667	0.435
	Urban	138	32 (23.2)	70 (50.7)	36 (26.1)		
**Level of knowledge**							
	Low	48	17 (35.4)	23 (47.9)	8 (16.7)	6.338	0.175
	Medium	108	24 (22.2)	59 (54.6)	25 (23.1)		
	High	38	7 (18.4)	18 (47.4)	13 (34.2)		
**Level of attitudes**							
	Negative	42	12 (28.6)	23 (54.8)	7 (16.7)	2.452	0.653
	Neutral	104	24 (23.1)	51 (49.0)	29 (27.9)		
	Positive	48	12 (25.0)	26 (54.2)	10 (20.8)		

* p value by Fisher’s exact test.

### Qualitative strand

The qualitative strand explored the benefits and barriers to involvement in HPS program among high school teachers. The personal characteristics of in-depth interviewees were summarized in Table 4.

**Table 4:** Characteristics of in-depth interviews among high school teachers by the level of involvement (n = 15).

IDI No.	Sex	Age	Education	Location of school	Level of involvement
HPS-01	Female	52	B.Ed.	Urban	poor
HPS-02	Female	36	B.Ed.	Urban	Poor
HPS-03	Female	56	B.Ed.	Rural	Poor
HPS-04	Female	51	B.Ed.	Urban	Poor
HPS-05	Female	24	B.Ed.	Urban	Poor
HPS-06	Female	28	B.Ed.	Urban	Medium
HPS-07	Female	29	M.Sc./M.Ed. and above	Urban	Medium
HPS-08	Female	43	B.Ed.	Urban	Medium
HPS-09	Female	40	B.Ed.	Urban	Medium
HPS-10	Male	25	B.Ed.	Rural	Medium
HPS-11	Female	46	B.Ed.	Urban	Good
HPS-12	Female	40	B.Sc./B.A.	Urban	Good
HPS-13	Female	29	M.Sc./M.Ed. and above	Rural	Good
HPS-14	Female	22	B.Ed.	Rural	Good
HPS-15	Male	21	B.Ed.	Rural	Good

#### School-based health literacy promotion

The majority of school teachers reported that by increasing health education activities in school, children could aware of health problems and improve their health knowledge. A small number of interviewees indicated that the students could share health knowledge with others. As one interviewee put it:

*“Improvement of health knowledge can provide the behavior change for healthy lives*… *Some parents can’t provide health education to their children well due to their low knowledge …*. *So*, *providing health education at the school can promote the level of health knowledge*.*"* (56 years old female teacher with poor involvement)

As a concern of the barriers, the majority commented that some students did not want to follow the teachers’ instructions or directives and they did not interest in some health education topics such as smoking, betel chewing, and substance misuse. Time constraint is another barrier for school teachers to provide health education. One participant reported:

*"Some children are in delayed growth…here*, *the one had a problem in hearing*, *and he can’t catch up with what the teacher taught*. *Although health education can improve the health knowledge of students directly*, *it may be better if it’s extended to the parents*, *caregivers and family members indirectly…*.*"* (24 years old female teacher with poor involvement)

#### Healthy environments

Clean classrooms, proper refuse disposal, water sanitation, and cleanliness of school toilets, healthy space for physical activities, and canteens were basic hygienic requirements for safe school environments. As the benefits, the high school teachers reported that school *environmental sanitation affected the physical and mental wellbeing of the students and a healthy learning environment*. Fresh in mind and keeping the focus in teaching could create a productive learning environment. Some interviewees suggested that a healthy school environment could help to improve concentration in learning. Commenting on the benefit of school environmental sanitation, one of the interviewees said:

*"We must establish our school as a garbage-free school*. *And we should involve in activities like spraying for insect and pest control and proper waste disposal to promote the positive image of the school… You know… It’s a benefit of health-related practice for the students not only during their school time but also for the time at home*.*"* (40 years old female teacher with medium involvement)

Concerning the barriers, insufficient human resources, low teachers’ involvement, and poor community participation were the limitations to involve in environmental sanitation. A small number of interviewees remarked that in the urban schools, the waiting for municipal garbage trucks to discard the wastes was the main problem in disposing of the refuse. One informant described:

*"Sometimes*…, *we can’t provide cleaning equipment such as hand diggers*, *rough broom*, *lawnmowers*, *etc*., *to the volunteers for doing school environmental sanitation*… *I mean… we have financial and human resource problems for doing these activities*. *So*, *we can do more for school environmental sanitation if we have efficient resources*, *both financial and human resources*.*"* (25 years old male teacher with medium involvement)

#### Prevention and control of both communicable and non-communicable diseases

The majority of interviewees suggested that dengue hemorrhagic fever, lymphatic filariasis, acute gastroenteritis, food poisoning, and worm infestations were the diseases that could be prevented by involving in school-based disease control activities. They believed that effective preventive measures in the school compound were crucial to prevent and reduce the spread of the diseases to the community. As one interviewee commented:

*“The preventive activities of communicable diseases are important to perform at the schools because the diseases can spread easily to other students at the school*. *If we check personal hygiene and health status of the students daily and the students also follow our instructions*, *the spread of diseases can be controlled*.*”* (43 years old female teacher with medium involvement)

As some difficulties, most interviewees commented that some obstacles such as low perception of students, poor participation of the students, and low family economic status affected the involvement of communicable diseases control activities. An interviewee commented:

*"It should be well-adjusted in the curriculum of teaching times and preventive activities of communicable diseases like checking personal hygiene and school canteen sanitation*. *The main difficulty is to argue the disobedient students to perform the disease control activities at the school*.*"* (56 years old female teacher with poor involvement)

#### Nutrition promotion and food safety

Nutritional promotion and food safety were the main activities to promote healthy eating habits and lifestyles of the students. *The main activities were checking for unhealthy food selling at the school canteens and providing health education related to healthy food*, *and the lunch box method*. The overwhelming majority of interviewees reported that eating nutritious food could not only promote immunity and physical growth but also diminish health problems. As one interviewee put it:

*"Sure…*, *we can get many benefits*. *Healthy children can learn better*. *The well-nourished students are more productive and can also create a healthy lifestyle in future*.*"* (40 years old female teacher with good involvement)

There were some suggestions that parent’s socioeconomic status and education level influenced on nutritional promotion activities of teachers such as providing nutritional knowledge and healthy school lunch box. A few interviewees indicated that some of the school canteen owners were poor in coordination with school health activities such as medical examinations for food handlers. Selling food and drink outside the school compound was also a challenge to food safety. One teacher stated:

*"Some students more prefer junk food or snack than the healthy food*. *And*, *it’s difficult to carry out the healthy school lunch box method because of the economic status of their families*.*"* (40 years old female teacher with good involvement)

#### School health services

The high school teachers supported the school health team in medical examinations such as primary oral care, personal hygiene, and BMI measurement of the students. The majority of interviewees reported that medical examinations in the school were checking the school canteens, measuring BMI, monthly vitamin supplementation, and transfer to the clinic when the students got sick. By ensuring medical examination as a pre-screening activity, diseases could be early detected. One participant reported:

*"It can support to reduce the chance of getting diseases and to be healthy students both physically and mentally*.*"* (52 years old female teacher with poor involvement)

It was possible to challenge the teachers’ involvement in the medical examination due to human resource limitation, time constraints, poor experience, and imbalance of the teacher-student ratio. Some schools did not have emergency first aid kit boxes for treating minor injuries. As one interviewee said:

*"As for me…*, *one difficulty is that some students don’t like examining their personal hygiene and… another one is the time limitation*. *So…*, *we can’t do it daily although we concern that it should be done daily*.*"* (24 years old female teacher with poor involvement)

#### Physical education, fitness, and sports

The majority of teachers commented that regular exercise could promote the physical development of the students, strengthen stamina, increase the circulatory flow, and refresh the mind and the body. Regular exercise and physical activities also helped the students to increase mental toughness and confidence to participate in collaboration or teamwork. Mental wellbeing, physical development, reduced stress and anxiety, minimize the risk of childhood obesity, and a sense of unity and teamwork were the benefits of doing regular physical activity. Another interviewee thought:

*"Regular physical activities help the children to achieve the physical and mental health*. *It promotes a sense of unity and enhances their educational performance*. *We want our students to create healthy behavior throughout their future lives*.*"* (21 years old male teacher with good involvement)

Regarding the difficulties of sports and physical activities, most of the teachers expressed that times for physical education were not enough for the students. Insufficient space for playgrounds, and limited material and human resources for physical education were also problems in carrying out this activity. One teacher stated:

*“In these days*, *some parents don’t allow their children to play outside or playground and want them to play indoor games to avoid getting an injury*. *So…*, *we are going to provide health education on awareness of sedentary lifestyle and its negative impact on health*.*”* (21 years old male teacher with good involvement)

#### Counseling and social support

Some interviewees reported that counseling and social support could assist to develop a positive attitude and solve the social problems of the students through parent-teacher cooperation. Moreover, the whole community might receive many benefits from healthy behavioral changes. It could promote the healthy behavior and communication skill of the students. One participant commented:

*"Children from broken families can have mental trauma*, *and we must do counseling to find the ways for dealing with emotional issues*. *I enforce the students to work hard and it is the main thing to achieve their goals*.*"* (56 years old female teacher with poor involvement)

As the barriers, the students could not follow the directives of the school health team and their parents could not cooperate with the teachers in the implementation of the school health activities. For a small number of interviewees, the students’ negative behavior, poor communication between students and teachers, and poor awareness of the students on the counseling activities were the main obstacles in successful implementation. One interviewee reported:

*"The language barrier is the main problem to carry out the counseling to the students who have varying cultural and ethnic backgrounds*.*"* (51 years old female teacher with poor involvement)

#### Community outreach

The majority of teachers replied that involving the community outreach to improve school health could be achieved by coordination with parents and community leaders. Community outreach of school health services could improve the effectiveness of life skill programs by changing the students’ negative school experiences and behaviors. It could also raise the parent awareness on health-related issues and opportunities to change for their healthy lifestyle. For example, one interviewee stated:

*"For the implementation of community outreach*, *we need to invite the parents*, *community leaders*, *and other administrative authorities to persuade in school health services*. *So*, *the positive behaviors could be adopted in the community by the initiation of school-based activities*.*"* (21 years old male teacher with good involvement)

Most teachers indicated that poor awareness of parents on school health services, low level of socioeconomic status of the parents, and lack of school health partnership by the community were the main barriers to involvement in community outreach. For the barriers on community outreach, one of the interviewees reported:

*"When we want the parents to come to the school for discussion the academic performant of their children*, *some parents gave a reason that they were working at that time or they couldn’t take a leave from the job*. *So*, *they couldn’t attend the Parent-Teacher meeting*.*”* (36 years old female teacher with poor involvement)

#### Training and research

The majority of interviewees suggested that updated information and skills regarding school health could be achieved by conducting research and training and they could precisely provide to the students in time. A small number of interviewees reported that involving in school health research was an activity finding practical ways for healthy behavior changes, improved social skills, and higher academic achievement for the students. As one interviewee said:

*"In the training*, *I got the opportunity of sharing and discuss the information that we haven’t known yet*. *Besides*, *I think we can report and advocate the difficulties to the upper level by doing researches related to school health promotion*.*"* (29 years old female teacher with good involvement)

Most of the teachers responded that as the difficulties for training and research, they have time constraints for conducting research, and some had poor skill and experience in doing research. Conducting an interview about this issue, an interviewee stated:

*“If I have a chance*, *I want to do the research related to dengue hemorrhagic fever*, *a common disease that mostly occurred during the rainy season*.*"* (21 years old male teacher with good involvement)

## Discussion

Schools play a particularly important role in establishing a safe and supportive environment with policies and practices to improve healthy behaviors among school children [36]. To prevent health problems and adopt a healthy behavior in the students’ daily life, the school teachers are a very significant role in the involvement of HPS program [37]. This mixed methods study aimed to assess the level of involvement and its associated factors as the quantitative strand and to explore the benefits and barriers to involvement in the HPS program among the high school teachers as the qualitative strand.

Among the 194 participants, most were female (94.8%) and the male-female ratio was 1:18. It was consistent with the studies done in Myanmar [26], Sudan [38], India [39, 40], and Nigeria [41, 42] stated that the majority of participants were females. However, this finding was contrary to previous studies conducted in Nigeria and Saudi Arabia [24, 43]. These differences could be due to differences in sociocultural and demographic backgrounds of study participants and variation in sample size. Currently, demographic, economic, political factors, and cultural norms were attributed to women for prevailing in teaching professions [44]. In Myanmar, teaching is one of the female-dominated professions and 86.2% of teachers in secondary education were female in 2018 [45]. Another reason for this was that males were household heads and teaching presents disincentives such as lower salaries, insufficient accommodation, and facilities [46].

Of all high school teachers, most of them (59.3%) were younger than 40 years and it reflected the finding of a previous Myanmar study, reported that the majority of participants were younger than 40 years [26]. The studies done in Sudan and Nigeria also stated that most of the participants were in the 30–39 years aged group [38, 43]. Another study showed that the majority of the teachers were aged 40–59 years [39]. In this study, the teachers with lower than 10 years of service duration were more than other categories and it matched the findings of earlier studies [26, 38, 47, 48]. However, it was contrary to that of Saudi Arabia study stated that the majority were 6 years and more services duration as teaching professionals [24]. Regarding the school location, urban school teachers were more than the rural school teachers in the recent study. It differed from some published studies, reported that rural school teachers were more than urban school teachers [26, 47, 49]. The possible explanations for these variations were due to the differences in the demographic nature of the study area and sample size of the study population.

School-based health programs could offer a highly effective platform for improving access to care and the health status of school-age children [50]. The school teacher could be an expedient source of health information for the students. In the current study, the findings indicate that 19.6% of high school teachers were those with a high level of knowledge regarding HPS program. It was lower than the findings of previous studies done in Myanmar with 62.9% of a high level of knowledge [26], Nigeria with 49.8% and 55.9% of good knowledge [51, 52], India with 40% of moderately adequate knowledge [47] and 22% of moderately adequate knowledge [48]. Conversely, it was higher than the findings of the other studies done in Nigeria with 8% of a good level of knowledge [43], 15.4% with adequate knowledge [53], and 16.3% with a good level of knowledge [41]. Unexpectedly, another study conducted in Nigeria reported that none of the participants had adequate knowledge of the school health program [42].

In order to make sincere efforts at implementing the HPS program, not only the program must be acceptable but also the teachers should have a positive attitude towards the program [41]. In the recent study, 24.7% of high school teachers had a positive attitudes towards HPS program and it was lower than the finding of the previous Myanmar study stated that the proportion of the school teachers with positive attitudes was 57.7% [26]. In Nigeria studies conducted among school teachers, 36.8% were aware of the school health program [51], 1.5% agreed that school health program would improve the academic performance of school children [41], and all head teachers from both private and public schools had favorable attitudes [42].

For the teachers’ involvement, the proportion of good involvement in the HPS program among high school teachers was 23.7% in this study. This finding was lower than the previous Myanmar study with 52.6% of high reported practices in specified school health activities [26] and Nigeria study with 47.1% of adequate school health activities [43]. However, it was higher than the another Nigeria study reported that 18.3% of heads of the school had good practices on school health services [52]. These inconsistent findings were might be due to differences in usage of an assessment tool for the level of knowledge, attitude, and involvement in the school health program, distinction of cutoff values in the scoring systems, and context variation of school health program depend on the geographical background.

This study confirmed that the age of the high school teachers was a significantly associated factor of good involvement in the HPS program. In accordance with this result, the previous Myanmar study had demonstrated that 50 years and older school teachers were more likely to had a good level of reported practices than younger school teachers and its association was statistically significant [26]. This result might be explained by the fact that older school teachers might have well experiences in school health services and more accomplished training related to school health services. However, this result had not previously been described in a study done at Southwest Nigeria [54].

In the current study, the high school teachers with 20 years and more duration of service were more likely to involve in the HPS program than the other categories of service duration. It confirmed the results of an earlier study conducted in Myanmar that duration of service was significantly associated with reported practices in school health activities among school teachers [26]. This also accorded with the observation of a Nigeria study, which showed that the teachers with a service duration of 21 years and more were more involved in health inspection of the students than those with less than 21 years of teaching experience [54]. There were some possible explanations for this result. Firstly, the school teachers with a long service duration could participate in the school-based health program with school health teams and might more concern about the health of the students based on their good experience.

Some of the factors influencing against effective implementation of the school health program were low levels of health knowledge and high levels of negative attitudes among the school teachers, and lack of resources [54]. The training programs related to school health services could be effective in situation analysis for assessing the level of knowledge and involvement in the program. In the recent study, the school teachers who accomplished three trainings regarding school health services were more likely to involve in the HPS program than those who accomplished one and two trainings. It was consistent with the previous Nigeria study stated that there was a significant relationship between previous formal training in the school health program and health inspection of the students [54]. The training focused on factual information related to school health services was critical for the school teachers to equip them with the necessary knowledge and might increase the involvement in the school health related activities [54, 55].

The school location was not an associated factor of involvement in the HPS program in this study. In contrast to earlier studies, it supported the evidence of previous Nigeria study [54] but differed from the findings of other studies approved that there was associated between school location and involvement of school teachers in the school health activities [26, 49]. The current study did not detect any evidence for the association of knowledge and attitude with the involvement of school teachers in the HPS program. School teachers, with their specific knowledge of students, can provide health knowledge, respond to specific needs of the students and create caring relationships that support the students’ well-being [54]. The current study found that level of knowledge and attitudes were not significantly associated with the involvement in the HPS program. These results matched those observed in the studies done in Myanmar and India [26, 49]. A Nigeria study also documented that there was no significant association between the level of knowledge and practices of school health services among heads of schools [52].

School health service is an appraisal of not only the contribution of health services to the school children and the members of the school family but also promoting health and providing security to the students [5]. The high school teachers believed that involvement in the HPS program could provide benefits to themselves, students, and the community such as improvement of the health knowledge, increase awareness of health problems in the school setting, the progress of healthy behaviors, improvement of physical and mental health, prevention of the disease spread, achievement of healthy and productive learning environment, and development of academic achievement.

School teachers could serve as reliable sources of health information and therefore, they could provide an appropriate vehicle for more integrated preventive health education [56]. A study conducted in Mandalay Region, Myanmar approved that providing health education is a great support to raise the knowledge of preventive measures of health problems among primary school teachers [57, 58]. Schools provide opportunities for the students to learn the practices of healthy eating and physical activity behaviors [37]. Food accessibility and availability influenced food choices and eating practices among the students. Consequently, creating a healthy environment plays an important role in their food choice during break time and school hours [58]. The students were more likely to maintain a physically active lifestyle by enhancing physical education and health education, and engaging families and communities in physical activity [59–61]. Moreover, physical activity could help in the improvement of academic achievement, and affect cognitive skills and academic behavior [62]. Therefore, the school teachers should implement the guidelines to optimize a coordinated approach to regular physical activity among school children, with the school health team, the media, religious organizations, and community organizations [37].

As regards community outreach, a Hong Kong study described that the schools under Healthy Schools Award scheme were more likely to be better school health policy, higher degrees of community participation, and better hygienic environment [63]. Community outreach in the schools’ activities is vital for the effectiveness of the HPS program and so, the school teachers should involve the school health activities closely with parents and the community [50, 64, 65]. In the benefits of the training and research activities, a qualitative study done in Bangladesh stated that the teachers’ training program related to school health had a positive impact on the ability, skills, and self-confidence of the teachers, who received training compared to the teachers who did not receive it [66]. Therefore, the school teachers should be accomplished a training program regarding specific school health activities.

While implementing the school health program, there were some challenges such as poverty’s effect on rural education, lack of resources especially in rural schools, lack of qualified teachers, transportation and communication problems, and low government investment [67]. In this study, insufficient materials and human resources, time constraints, incompetence of the teachers, poor cooperation of school health partnerships, insufficient awareness of parents, low family economic status were the barriers of the school teachers while involving in the HPS program. The contextual challenges including limited resources and inadequate infrastructure should be informed decision-making processes of how and what activities could be offered [68]. A qualitative study done in elementary schools from a school district in the Midwestern United States presented that lack of time due to increasing academic demands, lack of desire to play, peer pressure (especially in girls) not to be active and lack of space and equipment impacted the ability of students in physical activity [69]. In addition, increased time spent with social media and games, parents’ fears of safety for playing outside, and overscheduling of the students contributed to the poor participation in physical activity [70, 71].

The results of the current study should be considered in taking note of the strengths and limitations. Using mixed methods study was one of a strength of the study and consequently, the results could support to evaluate the in-depth insights of benefits and barriers to involvement in the HPS program among the high school teachers. It also revealed potential factors that influenced on implementation of school health services. However, the results of the study might be varied depending on the diversity of demographic (other States and Regions) and personal characteristics, although they might be generalized to those who were with the same background characteristics and setting. Additionally, the interviews were made only among high school teachers, and therefore, the results were in a lack of reflections by the primary and middle school teachers, headmaster or headmistress, school health officer, school nurses, school health partnerships, and community leaders regarding the involvement in the HPS program.

## Conclusion

The proportion of high school teachers with a high level of knowledge, positive attitude, and a good level of involvement in the HPS program were relatively low in the study area. Age, duration of service, and number of accomplished training regarding school health in high school teachers were associated with the level of involvement in the HPS program. As regards the benefits, the high school teachers believed that the HPS program could increase the health awareness of students and their parents, promote healthy behavior, diminish the health problems, create healthy and productive learning environments. This study suggested the key drivers that the major barriers to involvement of high school teachers in the HPS program were limitation of material and human resources, time constraints, lack of school health partnerships, poor coordination of parents and community, and low experience of teachers. It could be reduced by providing sufficient human resources and material, conducting on-the-job and refresher training to promote the skill, knowledge, and experience on school health activities such as providing health education, physical education, and research development, enhancing cooperation between school teachers and parents, and strengthening the continuation of school health services with the partnerships and community.

## Supporting information

S1 File Questionnaire. (PDF)

S2 File In-depth interview guideline. (PDF)

S3 File Minimal data. (XLSX)
