# Supplementary material for: Involvement of high school teachers in Health Promoting School program in selected township, Yangon Region, Myanmar: A cross-sectional mixed methods study
Source: PLoS One. 2022 Jun 16;17(6):e0270125. doi: 10.1371/journal.pone.0270125 (PMC9202905; doi:10.1371/journal.pone.0270125)
Supplement: S1 File — (PDF) [file pone.0270125.s001.pdf]

Quantitative Strand: Questionnaires

Code No. | | | |

Part 1. Personal characteristics

School location	☐ Urban	☐ Rural
Sex	☐ Male	☐ Female
Age ……………………………. years	Duration of service ……………… years	
Education	☐ B.Sc. (or) B.A.<br>☐ M.Sc. (or) M.Ed.	☐ B.Ed.<br>☐ Others ………………....
Accomplished training related school health (multiple response)	☐ Teacher Training Course<br>☐ Training for life-Skills Education<br>☐ Refresher Course in schools	

Part 2. Knowledge on health promoting school program

No.	Questions	Response		
		Yes	No	Don't know
**School-based health literacy promotion**				
1	Main health education topics at the high school level are -			
	(1) adolescent health	☐	☐	☐
	(2) oral hygiene & proper tooth brushing	☐	☐	☐
	(3) alcohol, smoking and narcotic drugs	☐	☐	☐
	(4) three main food groups	☐	☐	☐
	(5) sexually transmitted diseases	☐	☐	☐
2	Ways of health education are -			
	(1) discussion	☐	☐	☐
	(2) holding the trainings related with school health	☐	☐	☐
	(3) giving message by a study tour	☐	☐	☐
	(4) listening the health talk	☐	☐	☐
	(5) giving the health knowledge to students only	☐	☐	☐
**Healthy environments**				
3	Factors including school environmental sanitation are -			
	(1) school building and furniture	☐	☐	☐
	(2) excreta disposal	☐	☐	☐
	(3) school food stalls or canteen	☐	☐	☐
	(4) refuse disposal	☐	☐	☐
	(5) supply safe water	☐	☐	☐
4	Types of sanitary disposal methods are -			
	(1) buried	☐	☐	☐
	(2) composting	☐	☐	☐
	(3) discard on land	☐	☐	☐
	(4) discard into the river or stream	☐	☐	☐
	(5) municipal collection	☐	☐	☐
5	Safe drinking water is -			
	(1) pleasant of taste	☐	☐	☐
	(2) absence of color	☐	☐	☐
	(3) absence of harmful chemical substances	☐	☐	☐
	(4) absence of pathogen	☐	☐	☐
	(5) boiled water	☐	☐	☐

No.	Questions	Response		
		Yes	No	Don’t know
6	These hazards can get by the improper disposal of excreta -			
	(1) soil pollution	☐	☐	☐
	(2) contamination of food	☐	☐	☐
	(3) propagation of flies	☐	☐	☐
	(4) air pollution	☐	☐	☐
	(5) unsightly scene and public nuisance	☐	☐	☐
**Prevention and control of both communicable and non-communicable diseases**				
7	Activities of prevention and control of COVID-19 include -			
	(1) frequent hand washing with soap for at least 20 seconds	☐	☐	☐
	(2) wearing face-mask	☐	☐	☐
	(3) fever surveillance	☐	☐	☐
	(4) staying together of 3 feet from each others	☐	☐	☐
	(5) cover with fore-arm when coughing	☐	☐	☐
8	Diseases due to mosquito are -			
	(1) diarrhea	☐	☐	☐
	(2) malaria	☐	☐	☐
	(3) typhoid	☐	☐	☐
	(4) dengue hemorrhagic fever (DHF)	☐	☐	☐
	(5) filariasis	☐	☐	☐
**Nutrition promotion and food safety**				
9	The most effective way of giving knowledge on nutrition to is -			
	(1) school food stall or canteen	☐	☐	☐
	(2) donor	☐	☐	☐
	(3) lunch box	☐	☐	☐
	(4) parent-teacher association	☐	☐	☐
	(5) supervise the school canteen	☐	☐	☐
10	Nutritional ingredients containing daily eating food are -			
	(1) fat	☐	☐	☐
	(2) protein	☐	☐	☐
	(3) carbohydrate	☐	☐	☐
	(4) water	☐	☐	☐
	(5) minerals	☐	☐	☐

No.	Questions	Resonse		
		Yes	No	Don't know
**School health services**				
11	Daily checking activities of school medical examination are -			
	(1) cancers	☐	☐	☐
	(2) ear pus discharge	☐	☐	☐
	(3) common cold	☐	☐	☐
	(4) iodine deficiency disorders	☐	☐	☐
	(5) gynecological diseases	☐	☐	☐
12	Initial school medical examinations are -			
	(1) vision test	☐	☐	☐
	(2) hearing test	☐	☐	☐
	(3) oral health	☐	☐	☐
	(4) body development	☐	☐	☐
	(5) viral hepatitis	☐	☐	☐
**Physical examination, fitness, and sports**				
13	Standards of sport and physical activities are -			
	(1) honesty	☐	☐	☐
	(2) doing for self-interest	☐	☐	☐
	(3) justice	☐	☐	☐
	(4) respect to others	☐	☐	☐
	(5) obey discipline	☐	☐	☐
14	Advantages of doing regular physical activities are -			
	(1) strong muscles movement	☐	☐	☐
	(2) good circulation	☐	☐	☐
	(3) more deposit of fats	☐	☐	☐
	(4) increased body weight	☐	☐	☐
	(5) well mental alert	☐	☐	☐
**Counseling and social support**				
15	Ways of counseling and social supports are -			
	(1) greeting	☐	☐	☐
	(2) telling student about why they have to come	☐	☐	☐
	(3) helping on problem solving	☐	☐	☐
	(4) explaining about the needed facts for self- decision	☐	☐	☐
	(5) revisiting to the students for social support	☐	☐	☐

No.	Questions	Response		
		Yes	No	Don't know
16	Standard counseling methods are -			
	(1) needing private place	☐	☐	☐
	(2) needing enough time	☐	☐	☐
	(3) no caring of students' secrets and believes	☐	☐	☐
	(4) accepting of students' appraisal	☐	☐	☐
	(5) using difficult and un-understandable words	☐	☐	☐
**Community Outreach**				
17	Teams of community participation and healthy habits of school to outreach are			
	(1) parent-teachers association	☐	☐	☐
	(2) maternal and child welfare association	☐	☐	☐
	(3) school board of trustee	☐	☐	☐
	(4) school health committee	☐	☐	☐
	(5) red-cross society	☐	☐	☐
18	Ways of community participation and healthy habits of school to outreach are -			
	(1) telling forwarded plans of school to parents	☐	☐	☐
	(2) showing school activities to public	☐	☐	☐
	(3) doing medical services to parents' houses	☐	☐	☐
	(4) doing public benefit activities by students	☐	☐	☐
	(5) getting knowledge to parents by HE	☐	☐	☐
**Training and research**				
19	School health training -			
	(1) are delivered to headmasters by TEO	☐	☐	☐
	(2) are redelivered to teachers by headmasters	☐	☐	☐
	(3) of sub-school health tutor courses are delivered yearly in teaching training school	☐	☐	☐
	(4) must be discussed yearly at all school levels	☐	☐	☐
	(5) should be delivered in every township at least 3 times per year	☐	☐	☐
20	Conducting research activities -			
	(1) BMI examination	☐	☐	☐
	(2) doing other researches occasionally by sub-school health team in township level	☐	☐	☐
	(3) doing researches in parent's houses	☐	☐	☐
	(4) checking student-based school health activity	☐	☐	☐
	(5) doing health behavior researches of students	☐	☐	☐

Part 3. Attitude towards health promoting school program

No.	Statements	Answers				
		SA	A	U	D	SD
**School-based health literacy promotion**						
1	School health should be responsible only by health personnel.	☐	☐	☐	☐	☐
2	Teachers should give the school health education not only to the students but also to their parents.	☐	☐	☐	☐	☐
3	Prevention of DHF & oral hygiene should be the facts of health education to high school students.	☐	☐	☐	☐	☐
**Healthy environments**						
4	Poor hygiene of school food stall is one causal factor of infectious diseases in children.	☐	☐	☐	☐	☐
5	There should be at least one restroom for 80 students and not need to separate by gander.	☐	☐	☐	☐	☐
6	Harmful effects on health can be prevented by discarding the refuse away from the school.	☐	☐	☐	☐	☐
**Prevention and control of both communicable and non-communicable diseases**						
7	When the students is sick, teacher should refer to nearest health unit and inform to their parents.	☐	☐	☐	☐	☐
8	COVID-19 transmission should be prevented by hand washing.	☐	☐	☐	☐	☐
9	School canteen should be checked to prevent acute diarrhea that happens in students occasionally.	☐	☐	☐	☐	☐
**Nutrition promotion and food safety**						
10	School canteen cannot be affected by the school nutrition program.	☐	☐	☐	☐	☐
11	Lunch box method should be carried out at every school levels.	☐	☐	☐	☐	☐
12	The students should mainly eat the vegetables for physical growth.	☐	☐	☐	☐	☐
**School health services**						
13	The teachers must be prepared for the early detection of diseases of students.	☐	☐	☐	☐	☐
14	Teachers have no authority in medical examination of students.	☐	☐	☐	☐	☐
15	Oral health care should be carry out in primary education level only.	☐	☐	☐	☐	☐

No.	Question Items	Answers				
		SA	A	U	D	SD
**Physical education, fiteness, and sports**						
16	Physical training is doing within the school business time.	☐	☐	☐	☐	☐
17	Sport has no effect on a school going child of developing physical and moral powers.	☐	☐	☐	☐	☐
18	Regular physical exercise can prevent infectious diseases.	☐	☐	☐	☐	☐
**Counseling and social support**						
19	Academic counseling to students can be done by student themselves in addition to their parents and teachers.	☐	☐	☐	☐	☐
20	Educated counseling topics should remove from life-skills curriculums.	☐	☐	☐	☐	☐
21	Social supports are included in educated counseling and these also need the coordination of parents and teachers.	☐	☐	☐	☐	☐
**Community outreach**						
22	School Board Committee only has the responsibility for conducting community participation.	☐	☐	☐	☐	☐
23	Parents need to be invited when celebrating school health week.	☐	☐	☐	☐	☐
24	Development of school health function could be achieved by community outreach.	☐	☐	☐	☐	☐
**Training and research**						
25	Teachers should not do any research.	☐	☐	☐	☐	☐
26	Teachers should conduct the harm-related health behavior researches on students.	☐	☐	☐	☐	☐
27	BMI examinations of students are not included in research activity.	☐	☐	☐	☐	☐

SA: strongly agree, A: agree, U: uncertain, D: disagree, SD: strongly disagree

Negative statements: 1,3,5,10,12,14,15,16,20,22,24,26,27

Part 4. Involvement in health promoting school program

School-based health literacy promotion

1 Have you ever take the health education for personal hygiene?

☐ Yes

☐ No

2 Taking the health education for personal hygiene to -

☐ Both students and Parents

☐ Students alone

3 Which time you explain 10 facts of personal hygiene to the students?

☐ Daily

☐ Twice a week

☐ Weekly

☐ Twice a month

☐ Monthly

4 Have you ever take the health education for environmental sanitation?

☐ Yes

☐ No

5 Taking the health education for environmental sanitation to-

☐ Both students and Parents

☐ Students alone

6 Which time you explain environmental sanitation to the students?

☐ Daily

☐ Twice a week

☐ Weekly

☐ Twice a month

☐ Monthly

7 Have you ever check the personal hygiene status of students?

☐ Yes

☐ No

8 Which facts do you check in these activities?

☐ Brushing the teeth daily

☐ Clipping the hair daily

☐ Cutting the nail daily

☐ Cleanliness of cloths daily

☐ Washing the hand before eating and after toilet

Healthy environments

9 Have you ever check the cleanliness of classroom?

☐ Yes

☐ No

10 Which time do you check the cleanliness of classroom?

☐ Daily

☐ Twice a week

☐ Weekly

☐ Twice a month

☐ Monthly

11 Have you ever check the outdoor sanitation of school?

☐ Yes

☐ No

12 Which time do you check the outdoor sanitation of school?

☐ Daily

☐ Twice a week

☐ Weekly

☐ Twice a month

☐ Monthly

13 Have you ever check cleanliness of Cap and Cover by the student?

☐ Yes

☐ No

14 Which time do you check the cleanliness of Cap and Cover by the student?

☐ Daily

☐ Twice a week

☐ Weekly

☐ Twice a month

☐ Monthly

15 Have you ever check discarding of refuse away from school?

☐ Yes

☐ No

16 Which time you check discarding of refuse away from school?

☐ Daily

☐ Twice a week

☐ Weekly

☐ Twice a month

☐ Monthly

17 Have you ever check the cleanliness of toilets?

☐ Yes

☐ No

18 Which time you check the cleanness of toilets?

☐ Daily

☐ Twice a week

☐ Weekly

☐ Twice a month

☐ Monthly

Prevention and control of both communicable and non-communicable diseases

19 Have you ever clean the bush around the school for reducing mosquito biting?

☐ Yes

☐ No

20 Which time do you clean the bush around school for reducing mosquito?

☐ Daily

☐ Twice a week

☐ Weekly

☐ Twice a month

☐ Monthly

21 Have you ever guide to students about social distancing method of COVID-19 communicable disease?

☐ Yes

☐ No

22 Which time do you guide to students about social distancing of COVID-19?

☐ Daily

☐ Once in two days

☐ Once in three days

☐ Once in four days

☐ Once in five days

Nutrition promotion and food safety

23 Have you ever supervise the cleanliness of selling food items in school canteen?

☐ Yes

☐ No

24 Which time do you supervise the cleanliness of selling food items?

☐ Daily

☐ Twice a week

☐ Weekly

☐ Twice a month

☐ Monthly

25 Have you ever check the food-stall to sell the nutritious food?

☐ Yes

☐ No

26 Which time do you check the food-stall to sell the nutritious food?

☐ Daily

☐ Twice a week

☐ Weekly

☐ Twice a month

☐ Monthly

27 Have you ever take the school lunch programme in your school?

☐ Yes

☐ No

28 Which time do you take the school lunch programme in your school?

☐ Weekly

☐ Twice a month

☐ Monthly

☐ Two-monthly

☐ Three-monthly

School health services

29 Have you ever measure the height and weight of school children?

☐ Yes

☐ No

30 Have you ever record and report the height and weight of school children?

☐ Monthly

☐ Three-monthly

☐ Six-monthly

31 Have you ever check the medical examination of students?

☐ Yes

☐ No

32 Which time you check the medical examination of students?

☐ Daily

☐ Twice a week

☐ Weekly

☐ Twice a month

☐ Monthly

33 Which facts do you check the medical examination of students?

☐ Personal hygiene

☐ Seasonal communicable diseases

☐ Body development

☐ Vitamin deficiency disorders

☐ Skin diseases

Physical education, fitness, and sports

34 Have you ever train any physical activities to school children?

☐ Yes

☐ No

35 Which time do you train any physical activities to school children?

☐ Weekly

☐ Twice a month

☐ Monthly

☐ Six-monthly

☐ Yearly

Counseling and social support

36 Have you ever make counseling and social support to school children?

☐ Yes

☐ No

37 How many times do you make counseling and social support to school children?

☐ Less than two times

☐ Two to five times

☐ Six to nine times

☐ Ten to thirteen times

☐ More than thirteen times

Community outreach

38 Have you ever invite the parents when doing the school health committee?

☐ Yes

☐ No

39 Which time do you participate in the sessions of school health committee?

☐ Weekly

☐ Twice a month

☐ Monthly

☐ Six-monthly

☐ Yearly

Training and research

40 Have you ever receive yearly training of school health from teacher training college or headmaster?

☐ Yes

☐ No

41 How many times do you receive the school health training?

☐ Less than two times

☐ Two to three times

☐ Four to five times

☐ Six to seven times

☐ More than seven times

42 Have you ever accomplish any research activities concern with student's health?

☐ Yes

☐ No

43 How many times you do research activities concern with student's health?

☐ Less than two time

☐ Two to three times

☐ Four to five times

☐ Six to seven times

☐ More than seven times
