# Supplementary material for: Involvement of high school teachers in Health Promoting School program in selected township, Yangon Region, Myanmar: A cross-sectional mixed methods study
Source: PLoS One. 2022 Jun 16;17(6):e0270125. doi: 10.1371/journal.pone.0270125 (PMC9202905; doi:10.1371/journal.pone.0270125)
Supplement: S2 File — (PDF) [file pone.0270125.s002.pdf]

Qualitative Strand: In-Depth Interview Guideline

IDI No. HPS- | | |

Personal characteristics

Sex ______________________, Age ____________________ years

Education _______________________, Duration of service ___________________ years

School location _______________________, Level of involvement Poor / Medium / Good

Theme 1: School-based health literacy promotion

What activities are you being involved in ‘school-based health literacy promotion'? Why do you take the activities related school-based health literacy promotion?

What are the advantages of involvement in school-based health literacy promotion?

Probe: Get the students to know the health problem? Share the health knowledge from training?

What difficulties do you encounter?

Probe: No time for taking health education? Do not know how to take? No training for school health education? Think that do not need in high school level? Poor staff for taking?

Theme 2: Healthy environments

What activities are you being involved in ‘healthy environments’? Why do you take the activities related healthy environments?

What are the benefits of involvement in activities related healthy environment?

Probe: Get the students to know how to dispose refuse? Create the accident avoiding and safety place?

What barriers do you experience?

Probe: Think that it is not responsible by teacher? No time for taking? Do not know how to involve? Insufficient staff in school?

Theme 3: Prevention and control of both communicable and non-communicable diseases

What activities are you being involved in ‘prevention and control of both communicable and non-communicable diseases’ activities? Why do you take the activities related it?

What are the benefits of involvement in prevention and control of both communicable and non-communicable diseases?

Probe: Control the transmission to others? prevent the outbreak condition? Improve the health knowledge?

What barriers do you experience?

Probe: No knowledge in prevention and control of disease? No disease occurrence in students? No train for how to prevent and control of disease? Poor staff in school? No time for taking?

Theme 4: Nutrition promotion and food safety

What activities are you being involved in 'nutrition promotion and food safety'? Why do you take the activities related nutrition promotion and food safety?

What are the benefits of involvement in nutritional promotion and food safety?

Probe: Important in physical and mental health? Support to immunity of children? Improve the physical growth of children?

What difficulties do you encounter?

Probe: Think that only responsible by parents? Do not donation system? Poor participation of parent-teacher association? No training related this issue? No time for taking? Poor staff for taking?

Theme 5: School health services

What activities are you being involved in 'school health services'? Why do you take the activities related school health medical examination?

What are the benefits of involvement in school health services?

Probe: Important for physical health of the students? Early detection of health problems? To prevent the disease spread to others students and community? Early refer to health personals?

What difficulties do you encounter?

Probe: Do not know how to take? No training for school health services? No time to involve? Insufficient experience for involving?

Theme 6: Physical education, fitness, and sports

What activities are you being involved in 'physical education, fitness, and sports'? Why do you take these activities?

What are the benefits of involvement in physical education, fitness, and sports?

Probe: Promote the physical health? Make usual physical activities? Change to healthy behavior? Improve the physical strength?

What barriers do you experience?

Probe: No time to take the physical education? Do not know how to provide? Limited expert teachers for physical education? No space for physical activities and sports?

Theme 7: Counseling and social support

What activities are you being involved in ‘counseling and social support’ activities’? Why do you take these activities?

What are the benefits of involvement in counseling and social support?

Probe: Support the positive attitude of the students. Assess the social problems of the students? Improve the cooperation with the parents?

What barriers do you experience?

Probe: Time constraints for involvement? Do not know how to involve? Poor experience? Insufficient human resources?

Theme 8: Community outreach

What activities are you being involved in ‘community outreach’ activities? Why do you take these activities?

What are the benefits of involvement in community outreach?

Probe: By the instruction of headmaster? By the curriculum of school health week? Important in promotion of school health?

What difficulties do you encounter?

Probe: Poor participation of Parent-Teacher Association? Poor cooperation with community leaders? Constricted timetable with school lesson? Human resource limitation?

Theme 9: Training and research

What activities are you being involved in ‘training and research’ activities? Why do you take these activities related training and research?

What are the benefits of involvement in training and research?

Probe: Upgrade the knowledge regarding school health? Share the health knowledge from training? Assess the main problems of the students? Improve healthy behaviors? Improve cooperation with school health partnerships?

What barriers do you experience?

Probe: Poor knowledge in doing research? Poor experience in conducting the research? Time limitation to participate in doing research with school health team?
